# Comparing the Wave Characteristics of Breakdown Pulses of the Lightning Waveforms in the Himalayan Region

**DOI:** 10.1155/2021/6381439

**Published:** 2021-02-19

**Authors:** Pitri Bhakta Adhikari, Aashutosh Adhikari

**Affiliations:** ^1^Department of Physics, Tri-Chandra Multiple Campus, Tribhuvan University, Kirtipur, Nepal; ^2^Department of Electronics and Computer Engineering, Pulchowk Campus, Tribhuvan University, Kirtipur, Nepal

## Abstract

We have analyzed the breakdown pulse train with or without the main event in this paper. Among the selected 81 flashes, 36 flashes are starting positively, and 45 are starting negatively. Also, 58 flashes contain positive pulses, and 67 flashes contain negative pulses, whereas 44 flashes contain both positive and negative pulses. Among these 81 flashes, 22 flashes follow the main events, and the rest are isolated events. In this study, we got the main duration of PB pulses as 1.94 ms and the time interval between the breakdown pulse and return stroke as 61.49 ms. On taking each pulse train, we found the rise time to be 2.6 *μ*s, zero-crossing time 14.95 *μ*s, and the time interval between pulses 199.3 *μ*s. The largest pulse amplitude ratio in the preliminary breakdown pulse to the main event return stroke was 0.43.

## 1. Introduction

Lightning phenomenon is a common natural activity that occurs in the atmosphere. Lightning is the basic incident for the evolution of life on Earth. There is still very little information about the starting of the discharge phenomena within the thunderstorm. The breakdown process is the initiation of the discharging phenomena of all types of lightning, either cloud to cloud lightning or cloud to ground lightning [1–8]. The mechanism of the initiation of a cloud-to-ground flash occurs within the thunder cloud called the preliminary breakdown process. This initial breakdown occurs between the main negative charge center and the positive pocket charge in a thunder cloud. This breakdown process in the cloud to ground lightning is the initial phenomenon, and the PB pulses may be used as a measure of the strength of the positive charge pocket in the thunder cloud [2].

The preliminary breakdown process between the main negative charge center and the lower positive charge pocket occurs within 2 to 10 ms. It is followed by the stepped leader either immediately or within 400 ms [3]. The return stroke was considered to be preceded by electric field changes by the combination of the breakdown (B), intermediate (I), and leader (L) field changes. Among the BIL waveform, the breakdown (B) portion was interpreted by Proctor et al. (1988) as the start of the leader, while the intermediate (I) portion was found to occur sometimes only. If the initial breakdown is not a unique process, the beginning of the stepped leader and the intermediate portion of the BIL electric field change may be due to the negative leader's slowing as it encounters a positively charged region [5, 9]. The intermediate stage was due to the negative lightning charging of the vertical channel of the initial breakdown until the field at the bottom of the channel was high enough to launch a stepped leader [3].

The preliminary breakdown process pulses are reported to be bipolar, the initial polarity, in most of the cases being the same as that of the following return stroke pulse; however, some pulse trains with initial polarity opposite to that of the following return stroke was reported by Ogawa [10]. The amplitude of preliminary breakdown pulses observed in a temperate country Sweden was comparable to the following return stroke. In contrast, the amplitude of PB pulses was minimal in tropical country Sri Lanka [6]. Similarly, the PB pulses of winter negative lightning have a larger amplitude than the negative summer lightning found by Brook in 1922 [7].

The individual pulses of the PB pulse train have a total duration of 20–40 *μ*s, and the average rise time of the pulse is 10 *μ*s, recorded by Rakov et al. [11]. In 1979, Weidman and Krider reported that there are two or three smaller pulses superimposed on the rising portion of preliminary breakdown pulses. Still, in case of falling portion and opposite overshoot, preliminary breakdown pulses are smooth [8]. The preliminary breakdown pulses are much weaker in positive ground flashes in comparison with negative ground flashes. In the negative ground flashes, the PBP has more amplitude than return stroke in winter thunderstorm [12, 13]. However, the PB process radiates strongly in the positive cloud to ground flashes [14]. The values for both interpulse interval and total pulse duration of positive lightning were half of those of negative lightning flash [6]. The PB pulses amplitude is about 21 to 26 percent of the first return stroke in the summer thunderstorm in Sweden [15]. The preliminary breakdown pulses are seen for all types of flashes, either cloud to cloud or cloud to ground at higher latitudes, but it is not observed at lower latitudes. Generally, the PB pulses have less amplitude than the return stroke, but about 25% of the flashes contain a higher PBP peak than the return stroke [16]. The ratio of the PBP peak amplitude to that of the return stroke does not depend upon the distance of the lightning phenomenon from the point of measurement [16].

Nag and Rakov (2008) examined the preliminary breakdown pulse trains of the negative cloud to ground discharges, without following the return stroke waveforms. They categorized the preliminary breakdown pulse train based on the duration of the pulse train. About 46% of the pulse train has a short duration of 1 to 2 *μ*s called narrow pulses, whereas the remaining pulse train has large durations in 10 *μ*s called classical pulses [17]. Upward propagating negative leaders produce positive PB pulses, whereas negative PB pulses are produced by downward propagating negative leaders [18]. At Conghua in China, the preliminary breakdown pulses were about 11.6% of the total positive cloud to ground flashes. The amplitude of return strokes was about 6 times greater than that of the PB's peak amplitude pulses [19]. There are three different types of polarity based on the initial polarity of the PBP and return stroke: same polarity, opposite polarity, and composite polarity. In a higher latitude, the opposite polarity type is larger than that in lower latitude [20]. However, Marshall et al. (2013) agreed that the breakdown pulses are essential for each negative lightning flashes at any latitude [13].

In this research, the preliminary breakdown activity pertinent to the Himalayan thunderstorm and leading the main event such as return stroke and other events has been studied. The recorded waveforms of the activity of the signatures of preliminary breakdown pulses have been studied, analyzed, and compared with those of the pulses signature of previous studies.

## 2. Instrumentation

Measurement of lightning discharge phenomena has been used by different researchers, different methodologies, and different techniques. Among the different methodologies, the researchers used mainly photography, optics, electromagnetic and acoustics, or a combination of two or three of these. Among them, the measurement of electromagnetic fields is comparably easier and believable [21–23].

**Figure 1:**
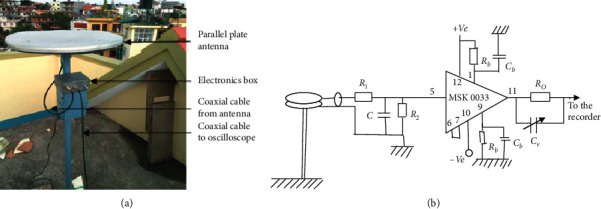
(a) The parallel plate antenna installed in Kathmandu; (b) the buffer circuit with antenna used in this research.

Due to the complex nature of lightning and practical limitations in mountainous regions, the electromagnetic field process is used here in this research. A parallel plate antenna is used to capture the vertical electric field signatures placed on the top of a building. The total height from the ground is about 13 m, and the altitude is 1300 m above sea level. The capacitance of the parallel plate antenna is 60 pF, and the output of the buffer amplifier was connected with Pico-scope 6404D (digital storage oscilloscope). Properly matched RG-58 coaxial cable is connected with the antenna, as shown in Figures 1(a) and 1(b).

The antenna and electronic system were carefully calibrated in the known electric field created by applying a voltage impulse from a Marx generator to a metallic grid plate. Galvan and Fernando have explained the details of the experimental measurement and calibration of the instrument [24]. In this paper, the digitizer volts are used to measure the electric field signatures. A similar electric field measuring system is described in various other experiments also [25–30].

## 3. Observations

**Figure 2:**
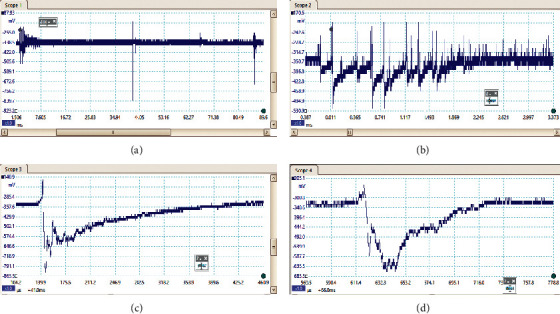
An example of several preliminary breakdown pulse train recorded and flash containing large number of pulses starting negatively and all the pulses are negative only and the preliminary breakdown pulses followed the return strokes.

A total of 81 flashes were selected to analyze the PB pulses from recorded a total of more than 150 flashes. Some were nondetectable and within the noise level, so only 81 flashes were selected. Among the 81 flashes, 36 flashes started from positive field change, and 45 flashes started from negative field change. On these 81 flashes, 22 flashes have other events preceded by PB activities, and the rest of them have isolated one. Out of these 81 flashes, 44 flashes were found to be bipolar pulses, whereas 58 flashes contained positive-type pulses and 67 flashes contained negative-type pulses. Only 14 flashes contained positive breakdown pulses only, and 23 flashes contained negative breakdown pulses only. These data are summarized in Table 1, and examples of different types of pulses are shown in Figure 2. The average duration of breakdown activity is found to be 1.94 ms with 5 pulses in positive flashes and 7 in negative flashes, on average. The minimum duration of breakdown activity is 0.3 ms, and the maximum duration is 8.47 ms, with a standard deviation of 1.5. The maximum number of positive pulses in a flash is 15, whereas the maximum number of negative pulses in a flash is 41. The standard deviation of positive pulses is 2.88, whereas for the negative pulses is 5.58. The peak amplitude of positive preliminary breakdown pulses is 271.1 mV on average, whereas the peak amplitude of negative preliminary breakdown pulses is 157.59 mV. The average interval between the breakdown pulses and the following events is 61.49 ms with a maximum time of 235.2 ms and a standard deviation of 71.59. These data are summarized in Table 2.

The mean time interval between the successive pulses is 199.3 *μ*s with the range of 1.8 *μ*s to 3.78 ms and a standard deviation of 311.7. The pulses' average rise time is 2.6 *μ*s obtaining a maximum of 41.6 *μ*s with a standard deviation of 4.2. Similarly, the pulses' zero-crossing time is 14.95 *μ*s on average, whereas the maximum zero-crossing time of the pulses is 582.8 *μ*s with a standard deviation of 48.6. The average amplitude of the pulse is 162.6 mV, obtaining a maximum of up to 1576 mV with a standard deviation of 191.2. The peak value of the amplitude of preliminary breakdown pulses in a flash is compared with the following main events on the same flash. The ratio of the peak value of the amplitude of preliminary breakdown pulses in a flash with the following main events return stroke is 0.43 on average. These data are summarized in Table 3.

## 4. Results and Discussion

The preliminary breakdown pulses in several published papers occurred with the field change prior to the other main events. Analyzing the electric field pertaining to ground flashes measured in South Africa reported by Clarence and Malan (1957) [3], the duration of field change was up to 200 ms, with 50% exceeding 30 ms and 10% above 120 ms. Kitagawa and Brook [31] also supported this value. Zhu et al. (2016) have reported a mean time interval of 8.8 ms between the PBP and the return stroke [32]. Gomes et al. reported that the interval between the most active part of the pulse train and the return stroke was 13.8 ms in Sweden and 11.9 ms in Sri Lanka. The mean interval of the preliminary breakdown pulses and the first return stroke is 27.7 ms reported by Sharma, with a geometric mean of 22.9 ms, a maximum value of 104.5 ms. The present study found that the interval between preliminary breakdown pulses and the first return stroke is 61.49 ms, with a maximum value of 235.2 ms and a minimum value of 4.34 ms. These values were obtained because of a small value of the amplitude of the PB pulses in this region. The average number of pulses per train found in this study is 6, slightly less than the average number of pulses per train found by Rakov et al. and Sharma et al. shown in Table 4. In this study, the observed pulse duration of 14.95 *μ*s is narrower than that reported by Rakov et al., 20–40 *μ*s, but wider than that reported by Sharma et al. 10.2 *μ*s. Similarly, the pulses' rise time in the present study is 2.6 *μ*s, which may be due to the Himalayan region and meteorological condition. It is shorter than that of Rakov et al. 10 *μ*s and Sharma et al. 5.2 *μ*s. However, the interpulse intervals or time between successive pulses of the preliminary breakdown pulses in this study are greater than those reported by Rakov et al. and Sharma et al., also presented in Table 4.

**Figure 3:**
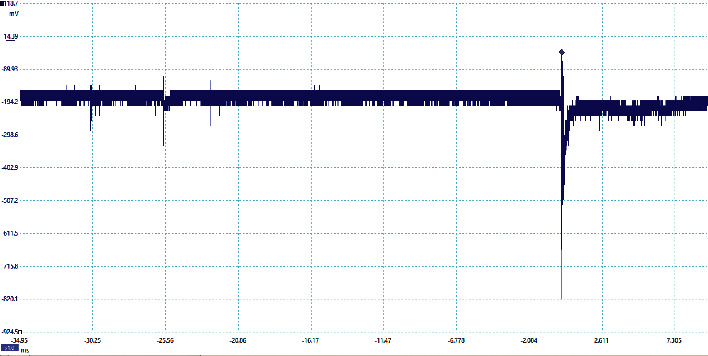
An example of the preliminary breakdown pulse train starting positively and followed the positive return stroke.

**Figure 4:**
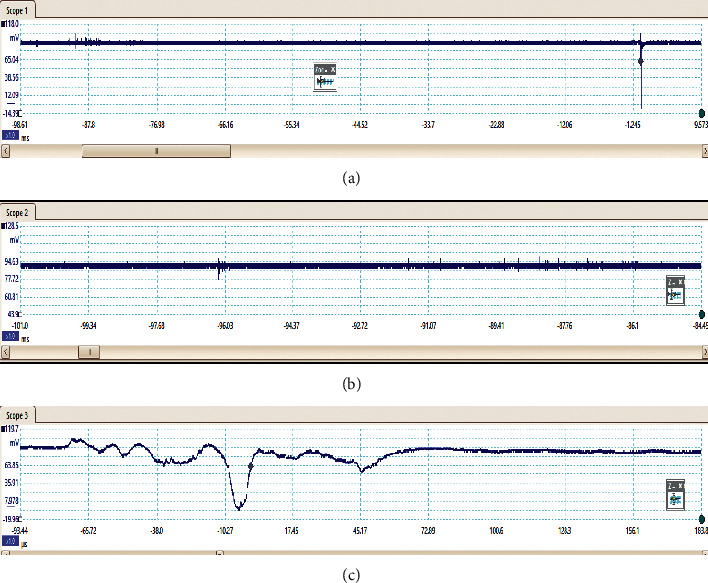
An example of the preliminary breakdown pulse train followed with the positive return stroke.

**Figure 5:**
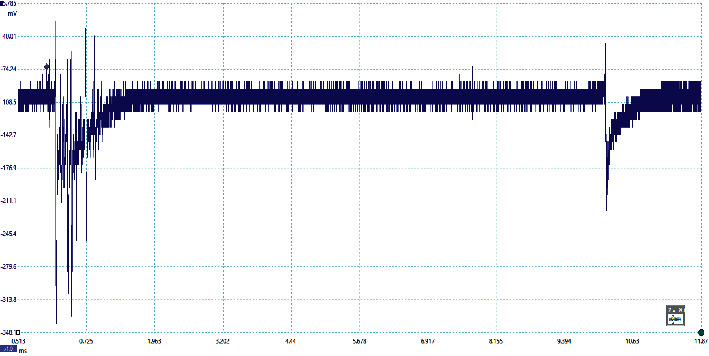
An example of the amplitude of breakdown pulses is nearly double than the amplitude of stroke.

**Figure 6:**
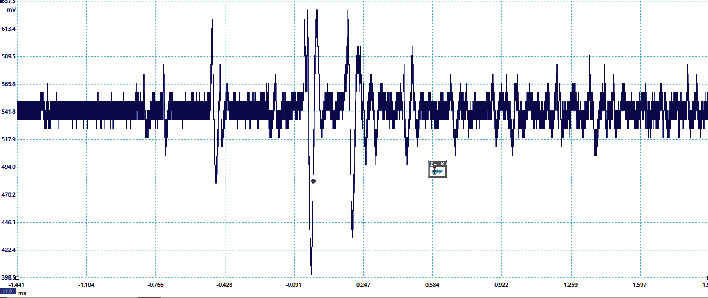
An example of isolated breakdown pulses in isolated form (IBP only).

The preliminary breakdown activity occurs in the lightning flashes due to the strong positive charge pocket on the cloud's bottom part. Due to the different meteorological conditions, the preliminary breakdown pulses occur at the initial stage of the thunderstorm activity and get weaker during the later stage of the thunderstorm. The signatures of some of the flashes observed in this study are preliminary breakdown pulses that occur before the main events, and some flashes have isolated breakdown pulses only, which are presented in Figures 2–6. The preliminary breakdown activity probably creates an ionized channel, which gives a path for the negative charge to progress towards the ground. Figure 2 shows an example of several preliminary breakdown pulse train recorded in the Himalayan region. This flash also represents a flash containing a large number of pulse trains, starting negatively with all pulse trains negative and the preliminary breakdown pulses following the return strokes. The time interval of the preliminary breakdown pulses and first return stroke is 37.45 ms, and the time interval of the first return stroke and second stroke and second and third strokes are 25.49 ms and 19.62 ms, respectively. Figure 2 shows the ratio of the amplitude of preliminary breakdown pulses and the amplitude of the first return stroke as 0.36, which is high compared to 0.15 of Zhu et al.  Figure 3 shows an example of a flash containing the preliminary breakdown pulse train starting positively, and the preliminary breakdown pulses followed the return stroke event. The time interval of the preliminary breakdown pulses and first return stroke is 22.6 ms.  Figure 4 shows an example of the preliminary breakdown pulse train with the return stroke recorded in the Himalayan region. The preliminary breakdown pulses followed the positive return stroke, whose time interval between the events is 79.33 ms. The ratio of the amplitude of the largest PB pulses to the amplitude of return stroke is 0.26. This means the amplitude of the largest PB pulse is 26% of the amplitude of return stroke. On the recorded waveforms of preliminary breakdown pulses, some of the pulses have an amplitude greater than the amplitude of return stroke. An example of this type of waveform is shown in Figure 5. As already mentioned, some breakdown pulses follow the main events. But in some flashes, only breakdown pulses are present, which are called isolated breakdown pulses. An example of an isolated breakdown pulse is shown in Figure 6.

**Figure 7:**
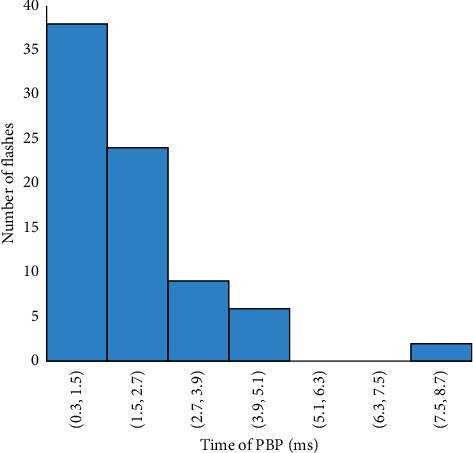
Duration of preliminary breakdown pulses (ms).

**Figure 8:**
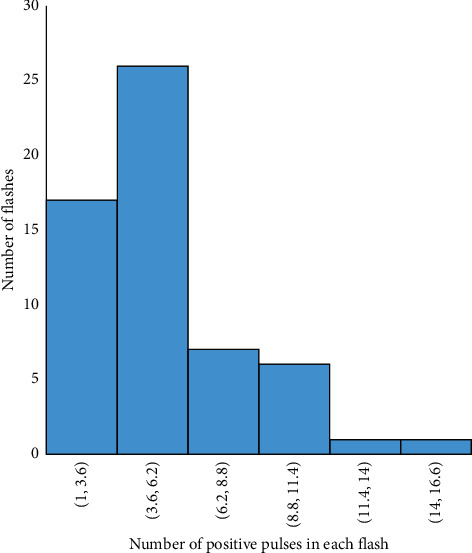
Number of positive pulses.

**Figure 9:**
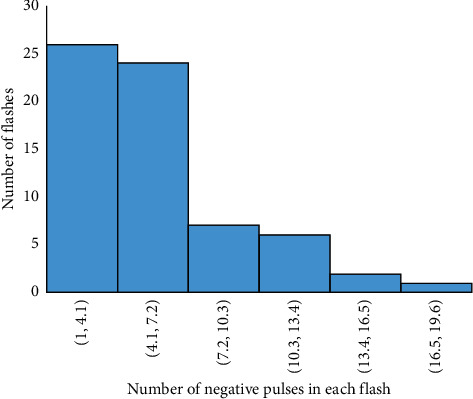
Number of negative pulses.

**Figure 10:**
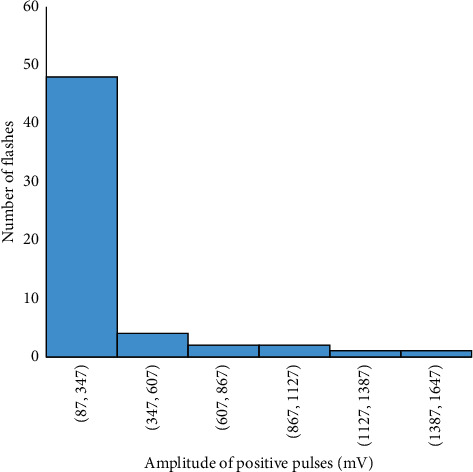
Peak amplitude of positive pulses.

**Figure 11:**
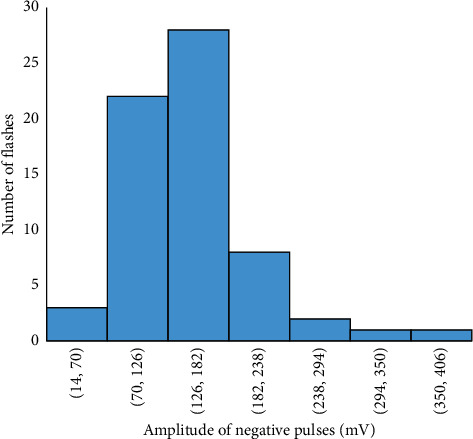
Peak amplitude of negative pulses.

**Figure 12:**
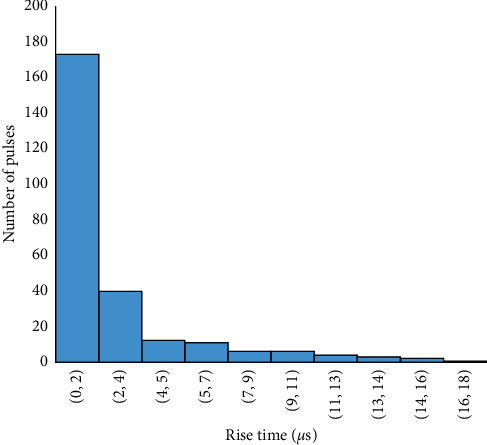
Rise time of preliminary breakdown pulses.

**Figure 13:**
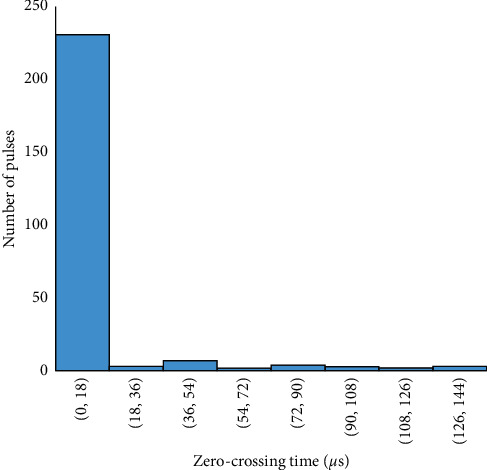
Zero-crossing time of preliminary breakdown pulses.

**Figure 14:**
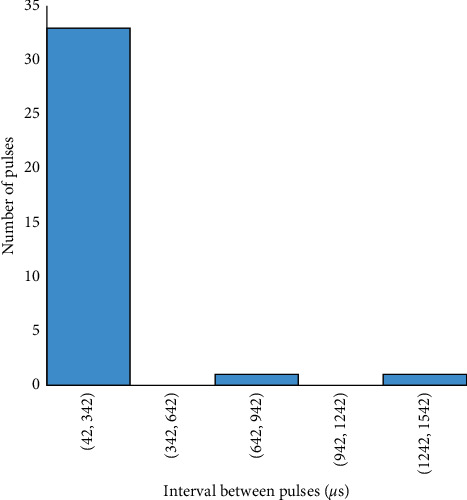
Time interval of preliminary breakdown pulses.

The frequency distribution of the duration of the preliminary breakdown pulse is shown in Figure 7. From this figure, 78.5% of the total flashes analyzed in this paper are below 2.7 ms, where the average duration of preliminary breakdown pulses is 1.94 ms. Upward propagating negative leaders produce positive PB pulses. In the positive PB pulses, there is positive electric field change. Similarly, negative PB pulses are produced by downward propagating negative leaders and have negative electric field change. Figures 8 and 9 represent the number of positive and negative pulses in which 74% of the positive pulses are below 6.2, whereas 75% of the negative pulses are below 7.2. Similarly, Figures 10 and 11 show the peak amplitudes of positive and negative pulses in which 83% of the positive pulses are below 347 mV, and 92.5% of the negative pulses are below 238 mV. 83% of the preliminary breakdown pulses' rise time is below 4 *μ*s, with an average of 2.6 *μ*s, as shown in Figure 12. Similarly, 89% of the preliminary breakdown pulses' zero-crossing time is below 18 *μ*s, with an average of 14.95 *μ*s represented in Figure 13. Also, 94.3% of the pulses has an interval below 342 *μ*s, which is illustrated in Figure 14.

## 5. Conclusion

The electric field radiated by lightning flashes over the rugged terrain of mountainous country Nepal was recorded and analyzed. The preliminary breakdown activity is pertinent to the Himalayan thunderstorm and leading the main events such as return stroke and other events. Some of the recorded waveforms were the signatures of preliminary breakdown pulses, and some were isolated. Both types of events have been studied, analyzed, and compared with previous other studies' pulses signature. The preliminary breakdown activity occurs in the lightning flash due to excessive positive pocket charges remaining on the bottom part of the cloud. The preliminary breakdown pulse phenomena depend on the amount of positive pocket charge remaining on the cloud. At the initial stage of the thunderstorm, the preliminary breakdown stage occurs, but these are weaker in the later stage of the thunderstorm in the Himalayan region.

## Figures and Tables

**Table 1:** Different number of flashes for different field changes.

Parameter	Number of flashes
Total	81
Flashes started from positive field change	36
Flash started from negative field change	45
Flash containing positive pulses	58
Flash containing negative pulses	67
Flash containing both polarity	44
Flash containing positive pulses only	14
Flash containing negative pulses only	23

**Table 2:** Different parameters of preliminary breakdown pulses in flashes.

Parameters	Duration of IBP/PBP (ms)	No of +ve pulses	No of −ve pulses	Peak amplitudes of +ve	Peak amplitudes of −ve	Interval of pulse and RS (ms)
Average	1.94	5	7	271.10	157.59	61.49
Maximum	8.47	15	41	1576	643.20	235.20
Minimum	0.30	1	1	86.66	14.2	4.34
Range	8.17	14	40	1489.34	629	230.86
Median	1.53	5	5	163.60	142.90	30.03
S. D.	1.50	2.88	5.58	282.51	91.99	71.59

**Table 3:** Different parameters of breakdown pulses.

Parameters	Rise time (*t*_*r*_) (*μ*s)	Zero-crossing time (*t*_*z*_) (*μ*s)	Amplitude (mV)	Time interval between pulses (*μ*s)	Ratio of amplitude of PBP with RS
Average	2.6	14.95	162.6	199.3	0.43
Maximum	41.6	582.8	1576	3784.8	1.89
Minimum	0.05	0.09	12.21	1.8	0.15
Range	41.55	582.7	1563.79	3783	1.74
Median	1.1	2.35	110.25	136.4	0.29
S. D.	4.2	48.6	191.2	311.7	0.47

**Table 4:** Parameters of preliminary breakdown activity compared to those from different geographical locations.

Field parameter	Rakov et al. [11]	Gomes et al. [6]	Zhu et al. [32]	Sharma [1]	Present paper
Duration of PBP (ms)	—	—	2.7	—	1.94
Time between PBP and RS (ms)	—	11.9 in Sri Lanka	8.8	27.7	61.49
		13.8 in Sweden			
No. of pulses per train	10	—	—	8	6
Pulse rise time (*μ*s)	10	—	—	5.2	2.6
Pulse duration (*μ*s)	20–40	—	25	10.2	14.95
Interpulse interval (*μ*s)	70–130	—	—	144	199.3

## Data Availability

The data used to support the findings of this study are available from the corresponding author, Dr. P B Adhikari, upon reasonable request.
